# A Partial Pulpotomy in Traumatized Permanent Incisors With Pulp Exposure

**DOI:** 10.7759/cureus.46432

**Published:** 2023-10-03

**Authors:** Soukaina El Kharroubi, Sofia Drouri, Bouchra Doumari, Kaoutar Laslami, Mouna Jabri

**Affiliations:** 1 Department of Conservative Dentistry and Endodontics, Faculty of Dental Medicine, Ibn Rochd University Hospital, Casablanca, MAR

**Keywords:** dental pulp exposure, follow-up, biodentine material, permanent incisor, partial pulpotomy, complicated crown fracture

## Abstract

A complicated crown fracture in a permanent incisor is one of the most difficult traumatic dental injuries to deal with. Treatment involves multiple visits and invasive intraoperative interventions and this is a very costly procedure. However, progress in vital pulp therapy and adhesive dentistry may allow practitioners to treat these injuries with a conservative method. Correct diagnosis of the pulp is important as it forms the basis for the establishment of an appropriate management strategy. A partial pulpotomy is indicated if the patient has significant pulp exposure or if it is reported after a considerable delay. It has been reported that partial pulpotomies after complicated crown fractures have a 96% success rate. Other studies have reported that partial pulpotomy is a treatment of choice after a complicated traumatic crown fracture, with a very high success record. Traditionally, calcium hydroxide has been applied as a dressing agent. However, research has recently focused on other calcium silicate cements (CSC), such as Biodentine (BD).

This study aimed to illustrate the successful management of a vital permanent incisor with complicated crown fractures, which were treated by partial pulpotomy using Biodentine material and evaluated for healing clinically and radiographically. No radiographic signs of failure or clinical symptoms were detected over a one-year period.

## Introduction

Dental trauma can have a profound impact on a patient's social and psychological well-being. Most cases of dental injury concern the anterior teeth, in particular the maxillary upper incisors. Crown fractures, with pulp exposure, represent 8.5% to 34.5% of all traumatic dental injuries. This is the most common trauma in permanent dentition, and its treatment in mature permanent teeth remains a challenge for clinicians. Root canal treatment has been the conventional option for mature teeth. However, guidelines published by the European Society of Endodontics (ESE) and the International Association of Dental Traumatology (IADT) currently suggest that complicated fractures (pulp exposure) of the crown of mature permanent teeth should be managed with vital pulp therapy, which is a conservative treatment that enhances pulp tissue healing and stimulates hard tissue formation to preserve pulp vitality, such as partial pulpotomy [1]. Unlike the conventional pulpotomy technique, which involves removing the entire coronal pulp, partial pulpotomy consists of removing only two to three millimeters of inflamed pulp to achieve healthy tissue and placing a biocompatible material that seals against microleakage. Conventionally, the material of choice has been calcium hydroxide (CH), but recent studies have shown that mineral trioxide aggregate (MTA) offers better marginal adaptation, reduces pulpal inflammation, and induces a thicker dentin bridge with less porosity [2]. However, despite its many advantages, MTA has certain drawbacks, such as a long initial setting time, difficult manipulation properties as a pulp capping material due to a wet sand-like consistency, and a risk of tooth discoloration [2]. This can be a major esthetic drawback, particularly on anterior teeth. To find an alternative product, research has recently been focusing on other calcium silicate cements (CSC), such as Biodentine (BD); it is a biocompatible and bioactive material that promotes pulp healing when applied directly in contact with pulp tissue [3]. The advantages of a partial pulpotomy over a complete pulpotomy include the preservation of cell-rich coronal pulp tissue, which offers greater recovery potential, and the continuation of dentin deposition in the cervical zone, which could otherwise be significantly weaker and more prone to fracture. The question arises as to whether pulpotomy should be the preferred treatment option for all pulps in teeth with complicated crown fractures.

The aim of this study was to determine and show the pulp outcomes of permanent mature teeth with complicated crown fractures treated with partial pulpotomy and using Biodentine as a suitable dressing agent and monitored clinically and radiographically for up to one year.

## Case presentation

**Figure 1:**
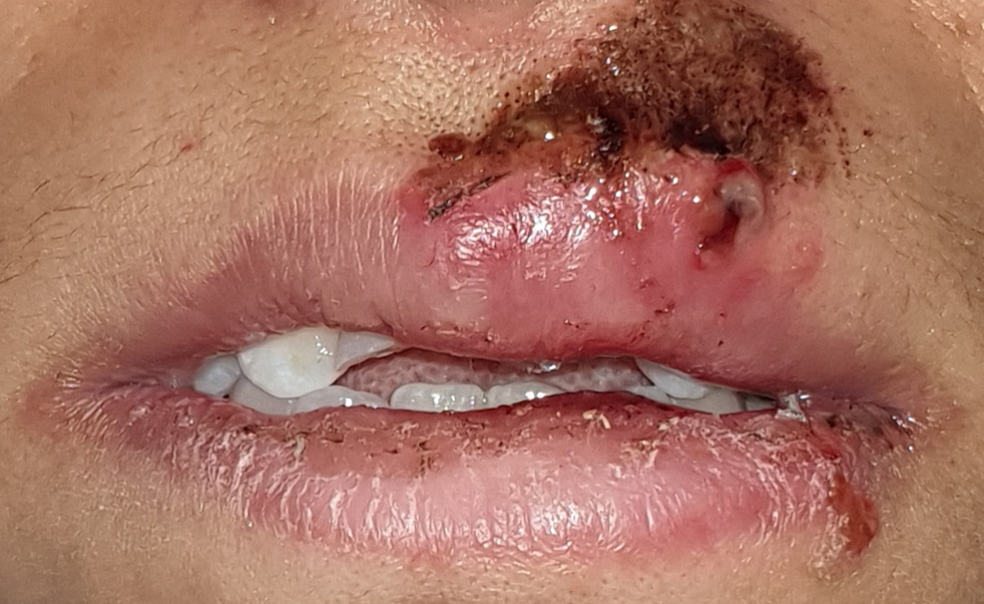
The presence of laceration injuries on the upper lip including swelling.

**Figure 2:**
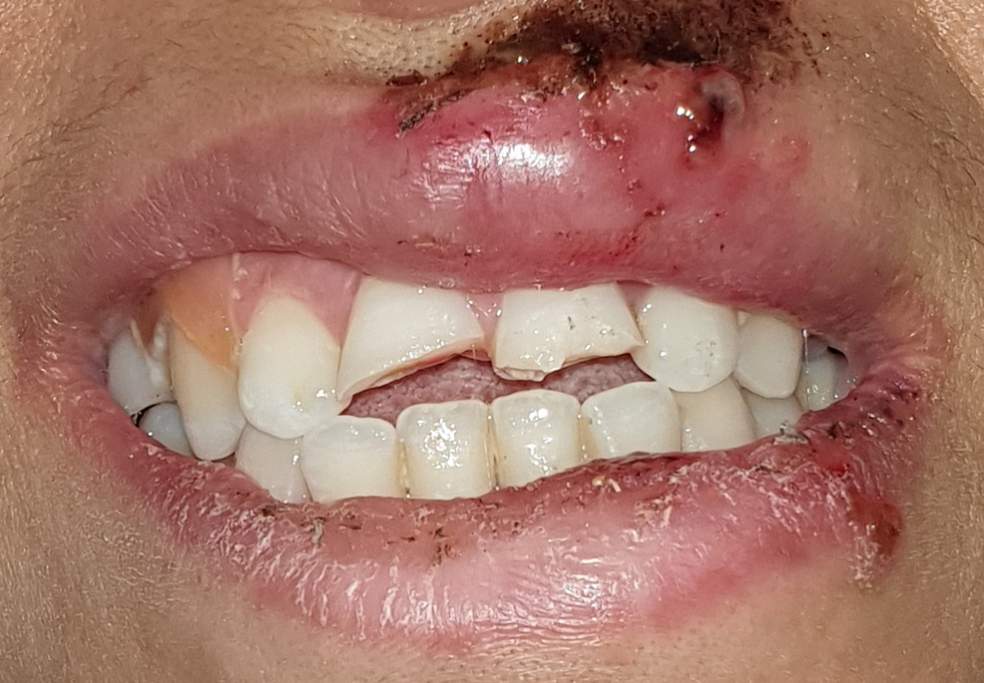
Traumatic injury to the upper front teeth.

A 28-year-old woman presented to the Department of Conservative Dentistry and Endodontics at the Centre de Consultation et de Traitement Dentaire (CCTD), Casablanca, Morocco, with a complaint of traumatic injury to the upper front teeth after a physical assault involving a fall to the ground. She sustained lacerations to the face and upper lip, as well as facial swelling and asymmetry (Figure 1). She was referred for treatment of her traumatic dental lesions 24 hours after the assault (Figures 1, 2).

**Figure 3:**
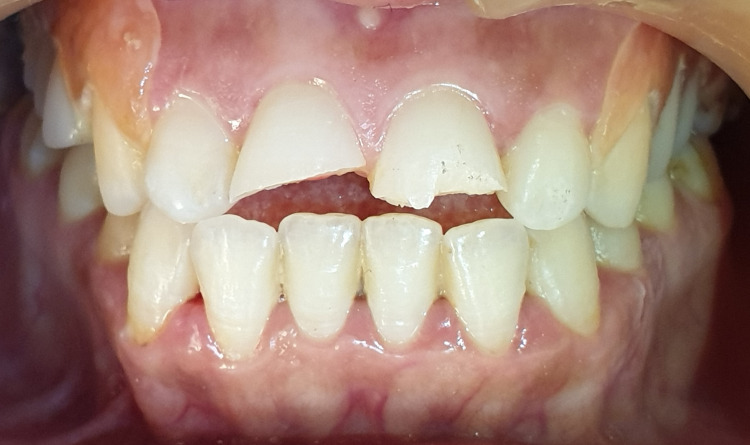
Intraoral examination showing the crown fracture to both incisors.

**Figure 4:**
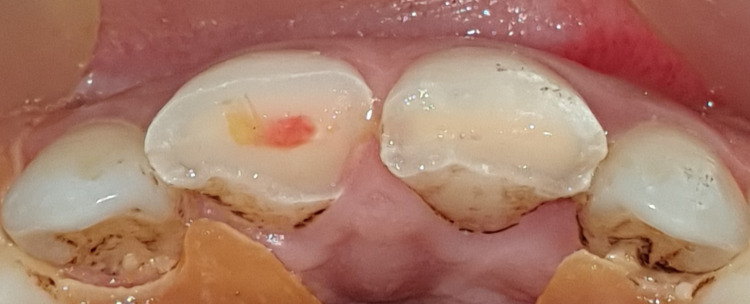
Complicated crown fracture with pulp exposure in tooth 11 and simple crown fracture in tooth 21.

The patient denied any spontaneous pain. Intraoral examination revealed a complicated crown fracture with pulp exposure in the right upper central incisor, and a simple enamel-dentin crown fracture without pulp exposure in the left upper central incisor, as well as mild pain on percussion, absence of pain on palpation, absence of periodontal pockets larger than 3 mm, mobility class I and a slight exaggerated positive response to the cold test on the labial surface of both incisors (Figure 3). The size of the pulp lesion was recorded in the upper right central incisor at almost 2 mm (Figure 4).

**Figure 5:**
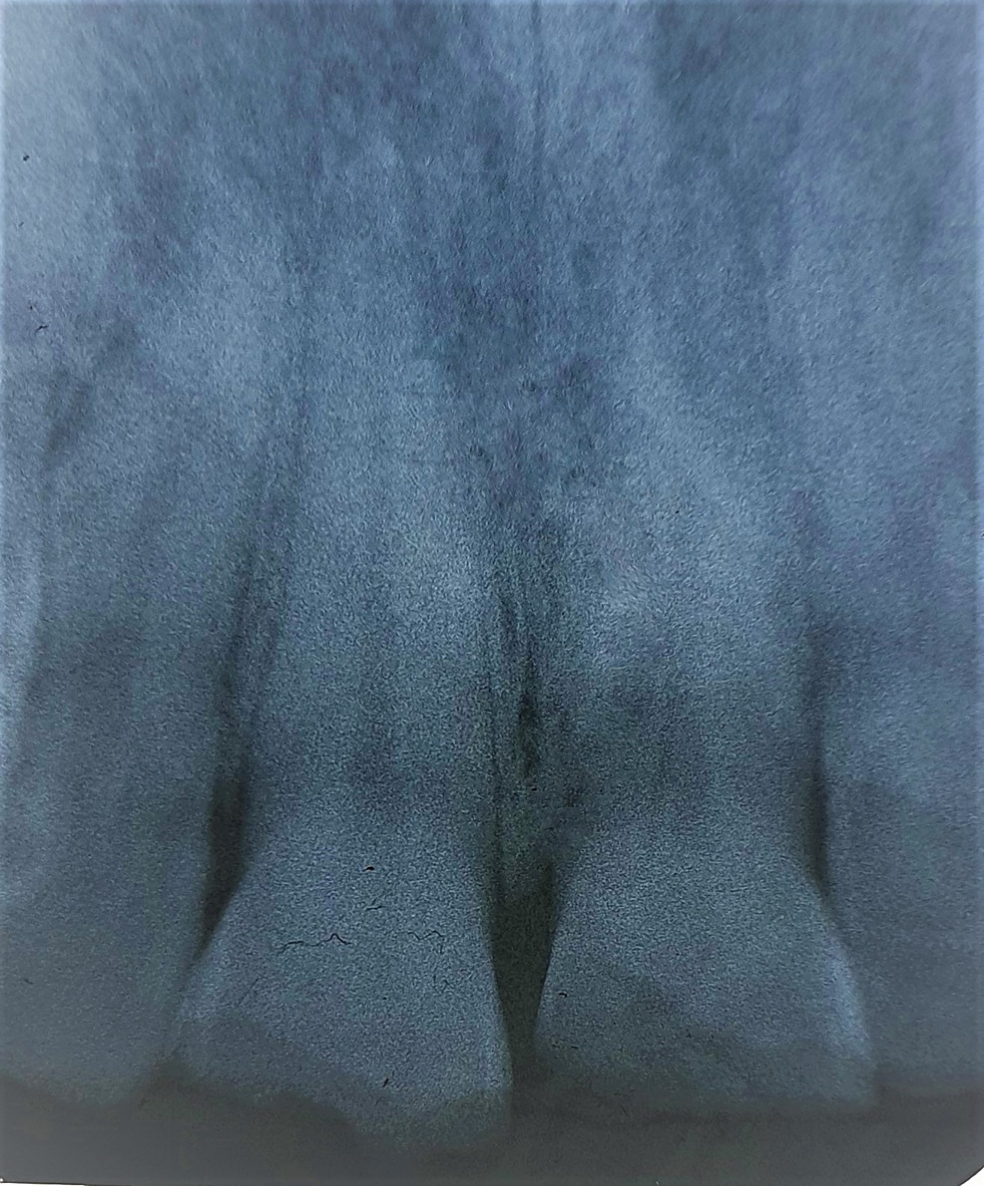
Radiographic examination validates the clinical observation and reveals the fracture line on teeth 11-21.

Radiographic examination confirmed the clinical observation and revealed a fracture line extending through the pulpal horns of the right upper central incisor and another fracture line distal to the pulpal horns of the left upper central incisor. Retroalveolar radiography provided sufficient and precise details of the fracture line in both incisors. Therefore, advanced imaging such as cone-beam computed tomography (CBCT) was unnecessary in this particular case. Fractures of both incisors not extending beyond the cervical line. No root fractures or peri-radicular radiolucency were detected (Figure 5).

**Figure 6:**
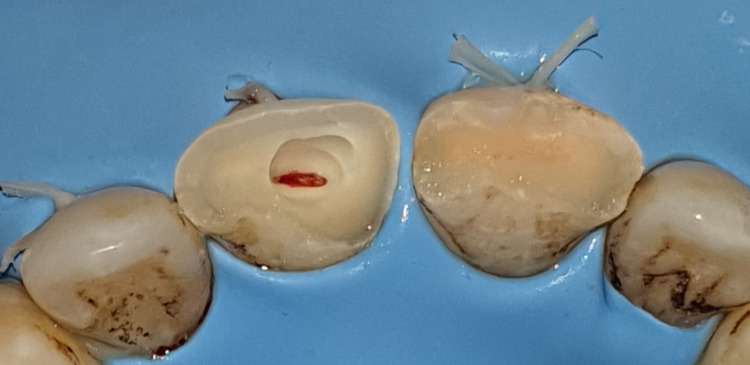
A 2 mm depth of visible pulp tissue and adjacent dentin of the right incisor was removed.

**Figure 7:**
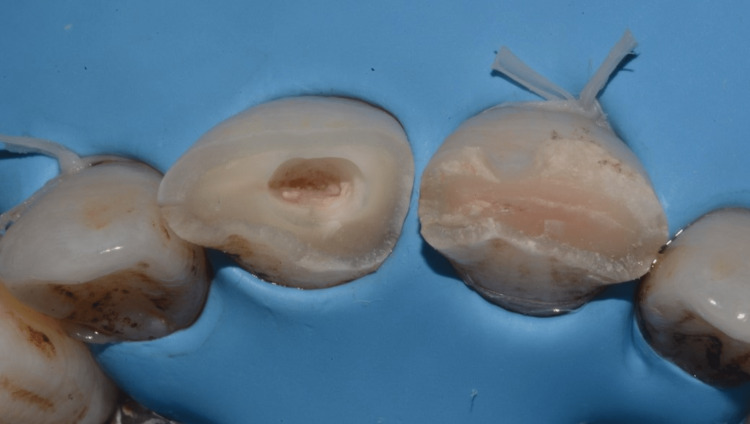
The bleeding was arrested after 5-10 minutes.

In this case, the patient was informed of the nature of the injury, and treatment options were also discussed. A treatment strategy consisting of a partial pulpotomy followed by a directly bonded composite restoration was decided upon and explained to the patient, who agreed to comply. After local anesthesia of the area involved, the teeth were isolated with a rubber dam, and almost 2 mm of visible pulp tissue and adjacent dentin of the right incisor were removed using a high-speed round burr and water irrigation. The pulp wound was gently rinsed with saline and cotton was placed to control bleeding (Figure 6). By removing the absorbent cotton after 5-10 minutes, the pulp was visible. Bleeding was arrested. Visual inspection revealed well-controlled bleeding and a healthy bright-red pulp. A hole approximately 2 mm deep remained, sufficient to contain the dressing and sealant (Figure 7).

**Figure 8:**
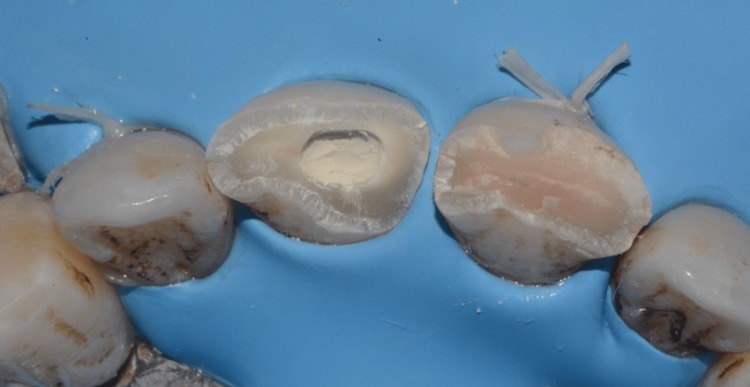
The exposed pulp surface was covered with a 2 mm thick layer of Biodentine on tooth 11.

**Figure 9:**
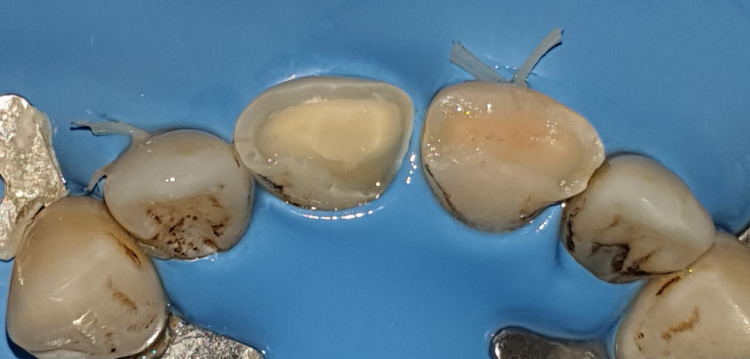
The Biodentine was covered with a layer of resin-modified glass ionomer.

**Figure 10:**
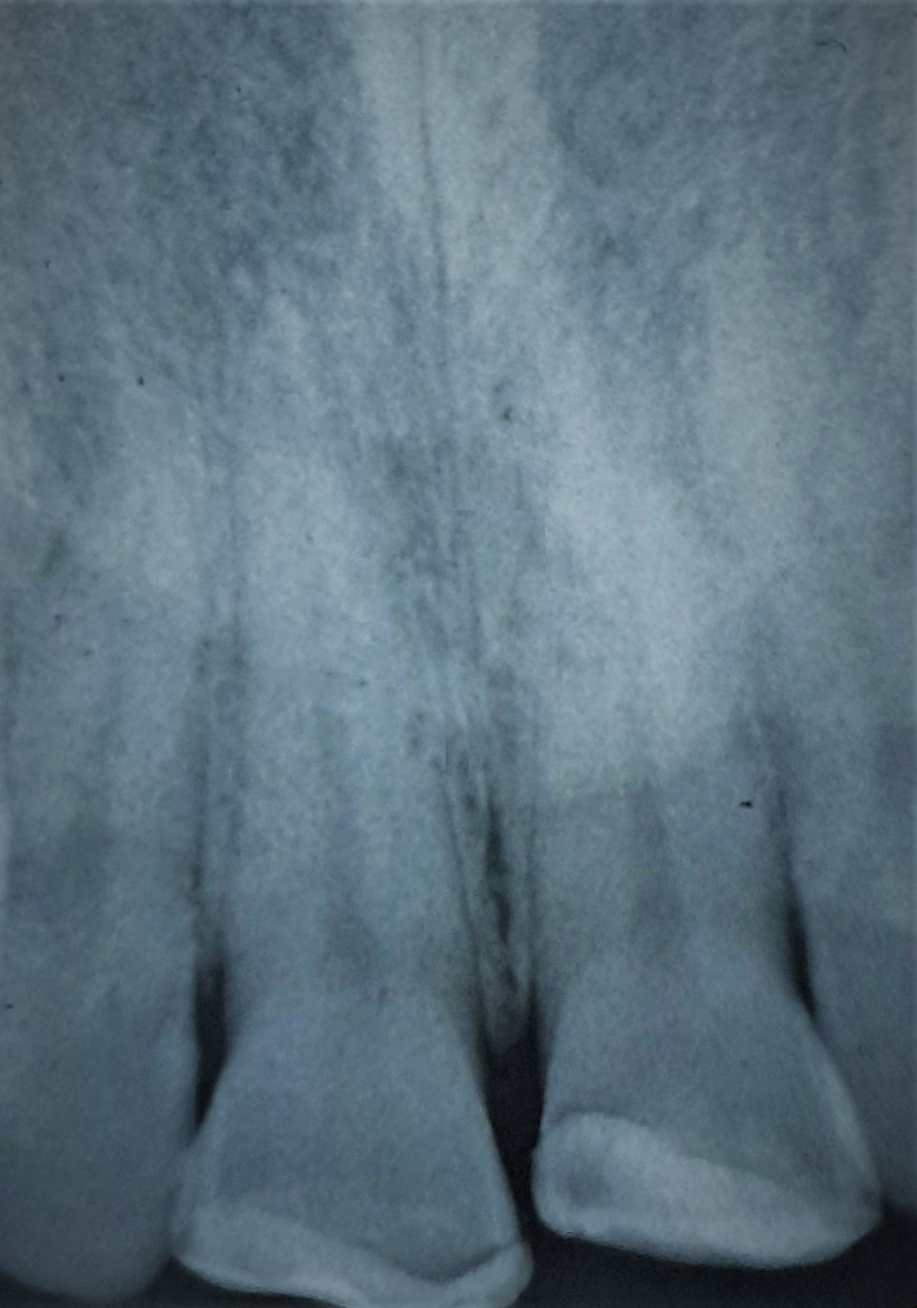
Layer of resin-modified glass ionomer is applied to teeth 11 and 21.

The exposed pulpal surface was covered with a 2 mm-thick layer of Biodentine, which was gently packed without pressure using moistened condensers and cotton pellets to fit the visible pulp space (Figure 8). The Biodentine was covered with a layer of resin-modified glass ionomer (Figure 9), which was also applied to tooth 21, and the patient was discharged (Figure 10).

**Figure 11:**
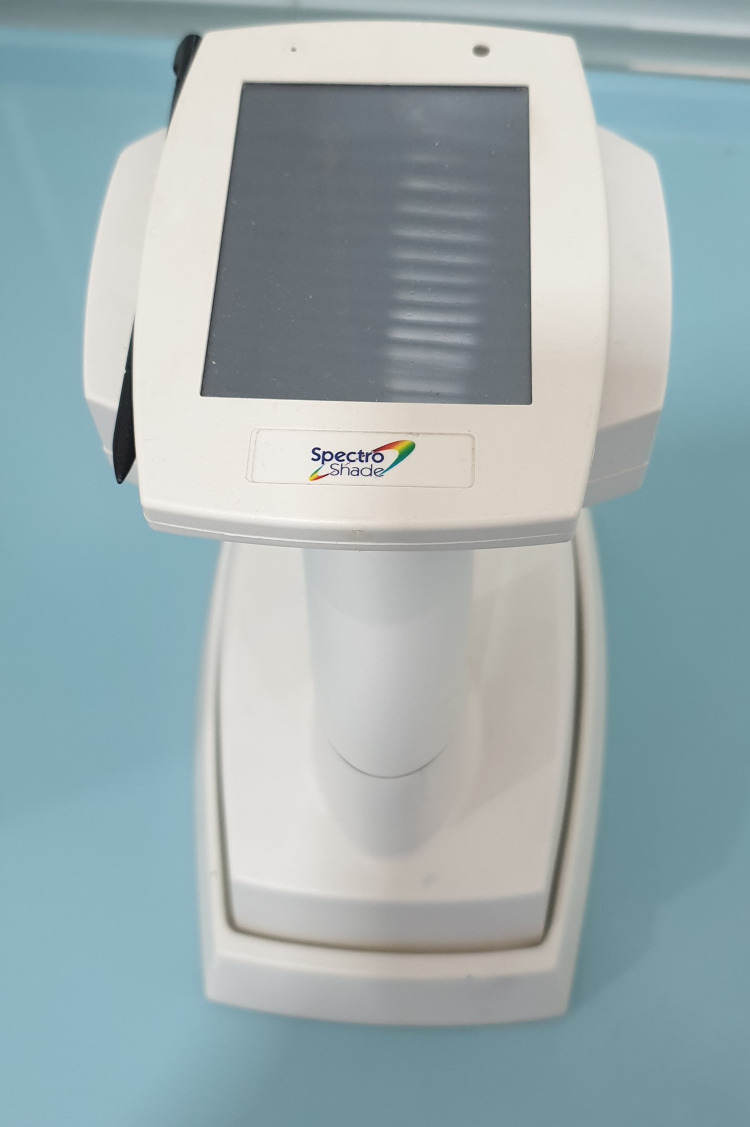
The shade of both teeth was chosen using SpectroShade Micro system. SpectroShade Micro system (Zürich, Switzerland: MHT Optic)

**Figure 12:**
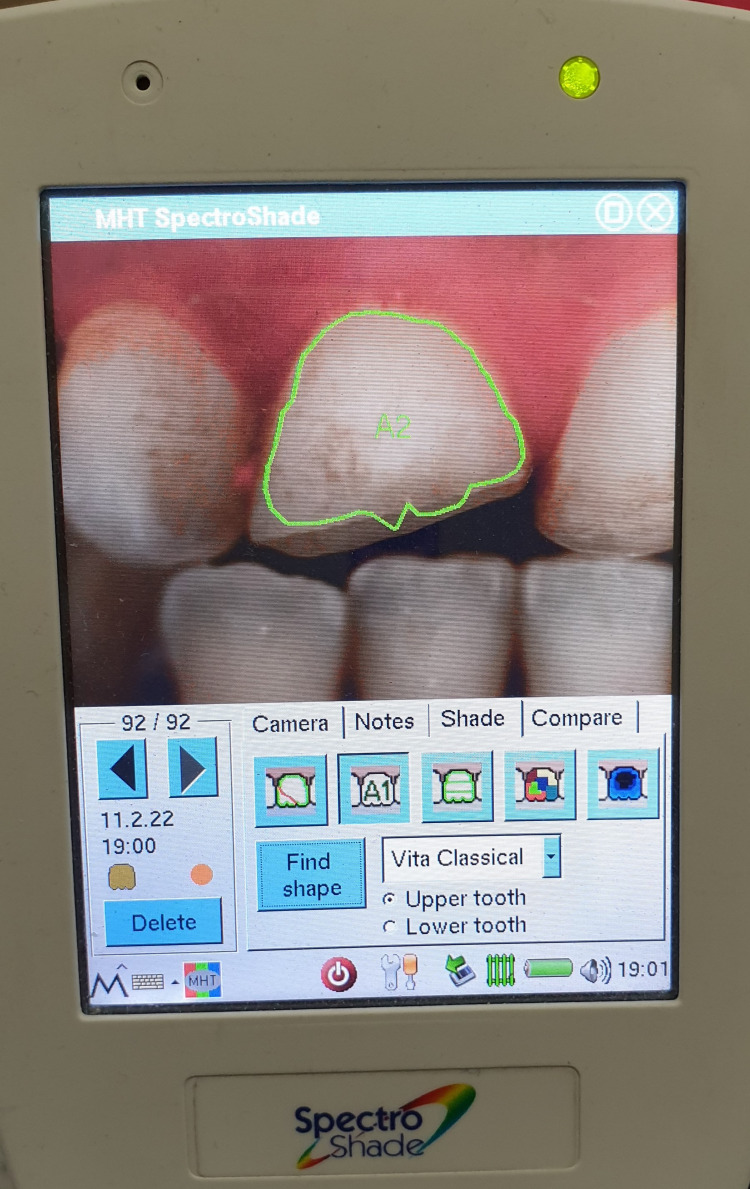
Several shade guide systems: A2/A3.

**Figure 13:**
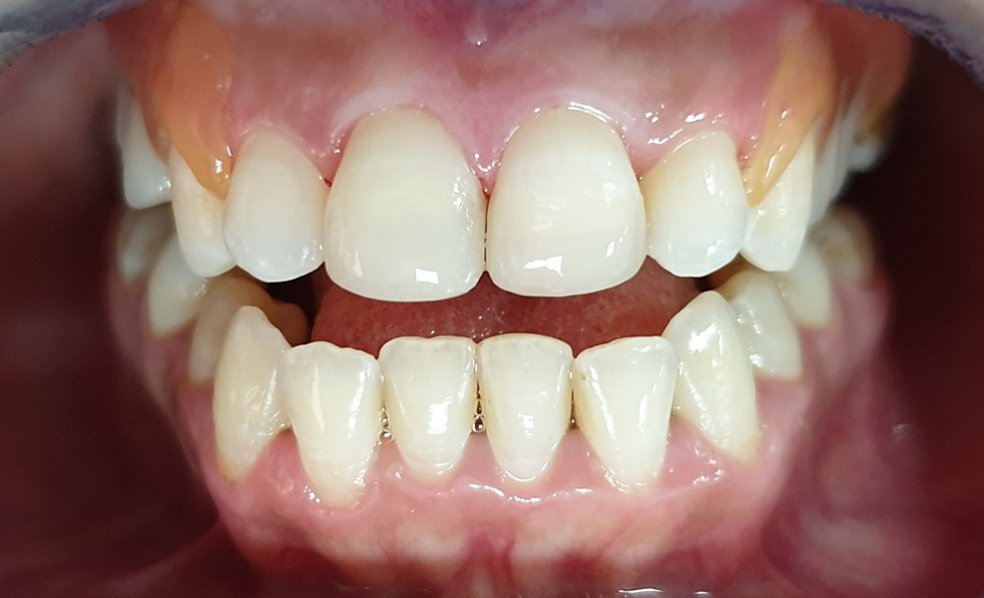
After one week, directly bonded composite restoration was performed on both teeth.

After one week, the patient was asymptomatic with a positive response to the cold test on both incisors. After selecting the shade of the two teeth using the SpectroShade Micro system (Zürich, Switzerland: MHT Optic) (Figure 11); it creates a color map that can be converted into several shade guide systems (Figure 12). The glass ionomer restoration was partially removed and a directly bonded composite restoration was made (Figure 13).

**Figure 14:**
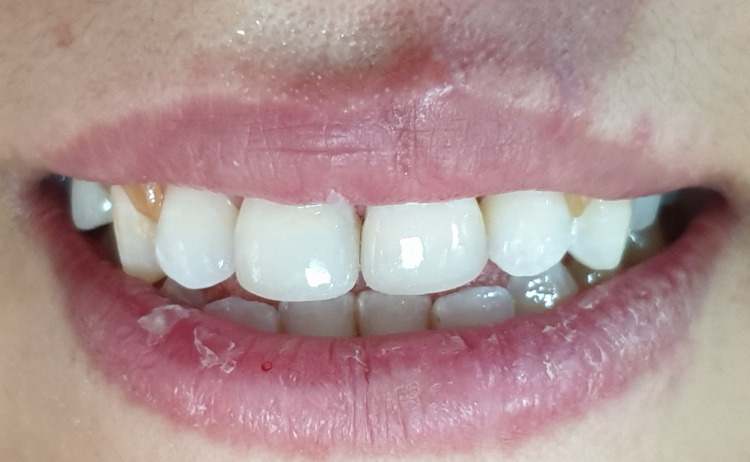
After one month of clinical follow-up.

**Figure 15:**
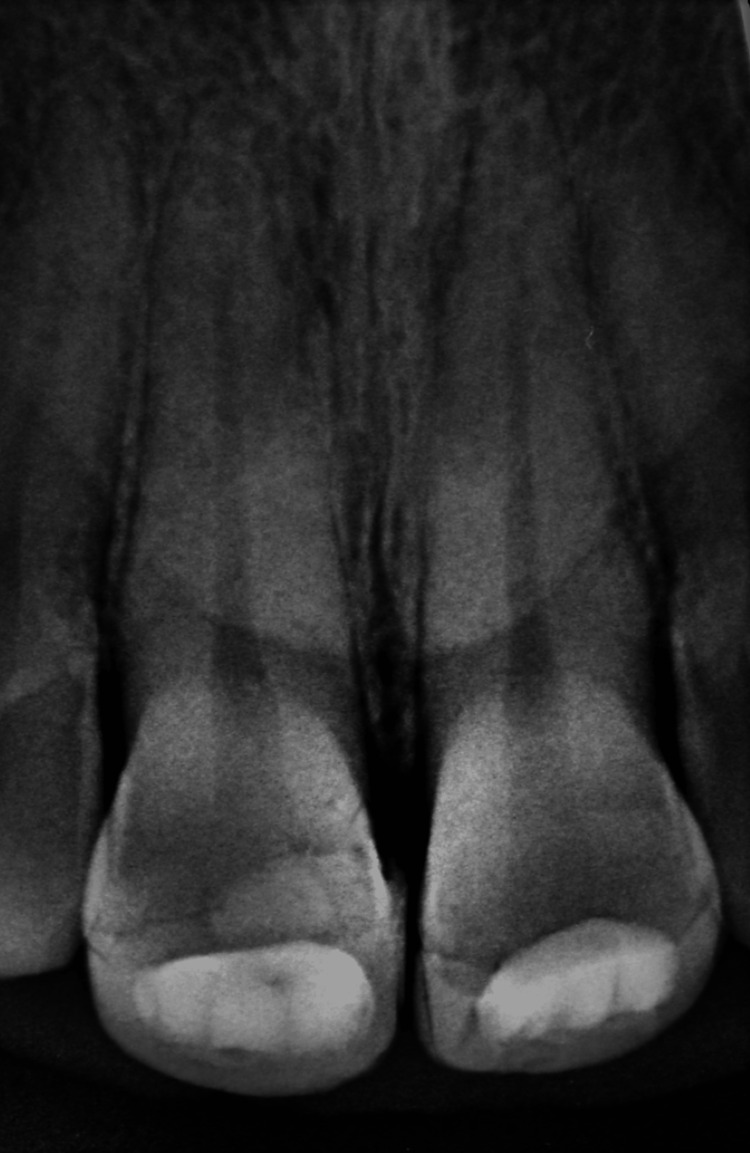
After one month of radiological follow-up.

**Figure 16:**
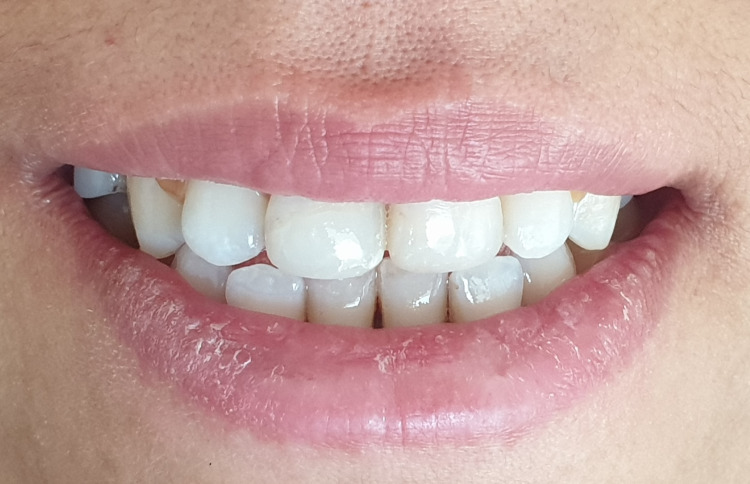
After one year of clinical follow-up.

**Figure 17:**
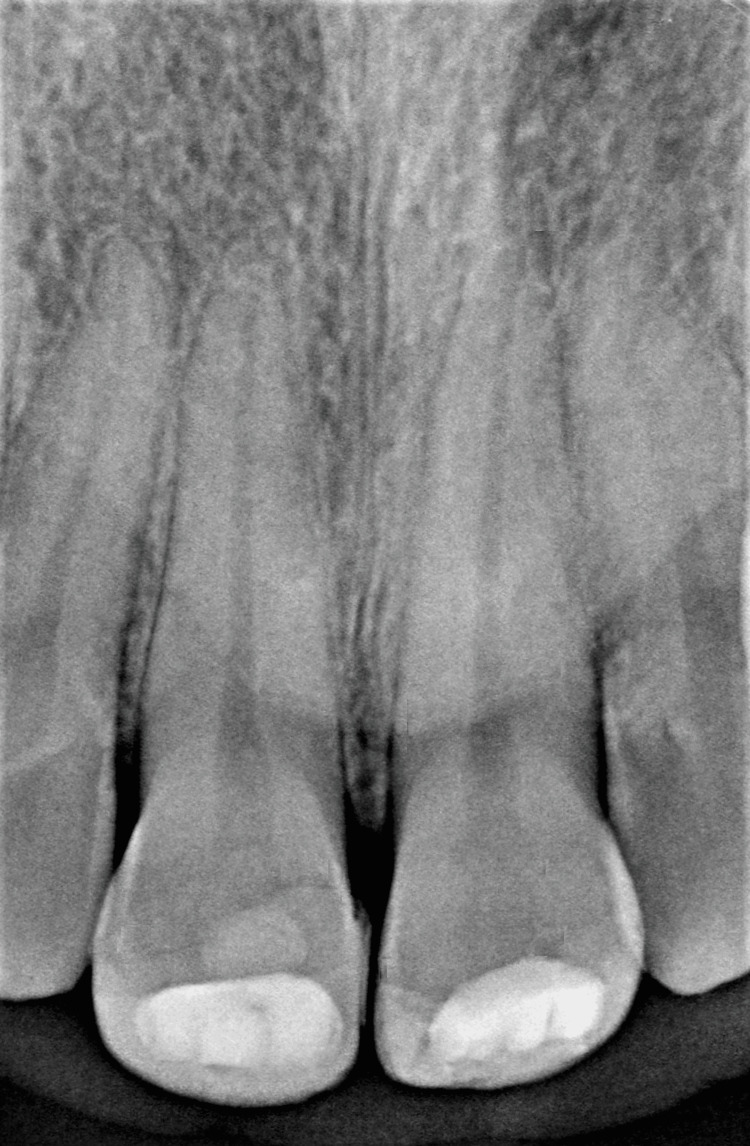
After one year of radiological follow-up.

The patient was recalled at two weeks, one month, six months, one year, and annually to check pulpal vitality, periodontal tissue health, and composite restoration. The patient's one-year follow-up revealed an esthetic and functional crown. Teeth were monitored clinically and radiographically (Figures 14, 15). The situation remained stable with a positive vital response to the cold pulp test, indicating that the pulp is undamaged and safeguarded. Radiographs showed normal peri-root integrity with no evidence of apical periodontitis or necrosis (Figures 16, 17).

## Discussion

Dental trauma is a frequently encountered problem in dental practice. The largest number of traumatic dental injuries in the permanent dentition involves around 80% of maxillary central incisors and 16% of lateral incisors. There are various classifications of traumatic dental injuries [1]. Complicated crown fractures affect the enamel and dentine, exposing the pulp. Approximately 18-20% of dental traumas result in crown fractures with pulp exposure. The management of traumatic dental injuries is a major challenge during treatment, which must prioritize the preservation of natural dental tissue, as lost tissue cannot be regained. The treatment strategy applied is based on the notion of minimal intervention and maximum biological tissue preservation [4]. In recently updated guidelines published by the International Association of Dental Traumatology (IADT), the focus has been on conservative pulp therapy (CPT) as the most effective treatment option for traumatized vital teeth with pulpal exposure, with the primary goal of maintaining pulpal vitality [1]. Several variables, including vital pulp therapy technique, hemostatic/irrigation agent used and competence of the practitioner, pulp capping material, size of pulp exposure, the time interval between trauma and vital pulp therapy (VPT), and final coronal seal, coexisting trauma, stage of root development, pulp contamination, appear to have an influence on the prognosis of traumatized teeth with pulp exposure. However, the evidence regarding some of these factors is sometimes conflicting [5].

Vital pulp treatments

The treatment options for VPT of traumatized vital permanent teeth are direct pulp capping, complete pulpotomy, and partial pulpotomy. The success rates for these treatments are 54.5-81.5% for direct pulp capping, 86-92% for coronal pulpotomy, and 94-96% for partial pulpotomy. The partial pulpotomy is defined as "a procedure that requires the amputation of the coronal portion of the damaged and hyperemic pulp tissue and dentin surrounding the exposure, with the use of a sterile round diamond bur in a high-speed handpiece, and a light touch and generous saline irrigation [6]. A 2-3 mm of the damaged and inflamed superficial pulp tissue is surgically removed to the level of the healthy pulp. Hemostasis is carried out using sterile cotton pellets for 5 min. Once bleeding has stopped, the exposed pulp is dressed in a biocompatible material to enhance healing and maintain the vitality of the residual pulp tissue [6]. Recent studies have shown that as long as a good hermetic seal is ensured, further root canal treatment is not necessary. The natural color and translucency of the tooth are preserved, which maintains the vitality of the pulp, and sensitivity testing is possible. This has also been confirmed by histological examinations and, over the years, the literature has continued to support this evidence [1]. In a 2022 study by Radwanski et al., it was shown that due to the partial pulpotomy's high success rate ranging from 86% to 100%, it is considered the treatment of choice for traumatic pulpal exposures [5]. Furthermore, in a study by Wang et al. in 2017, the pulpal necrosis rate after treatment of 375 teeth with complicated crown fracture was considerably higher for teeth that had been managed by pulp capping compared with partial and complete pulpotomy [7]. More than half (57.1%) of pulps treated with direct pulp capping suffered necrosis, compared with 10.1% for partial pulpotomy. Another study by Cvek et al. in 1982 revealed that complicated crown fractures treated by partial pulpotomy with a treatment time of 1 hour to 90 days had a success rate of 96% [8]. The results of many recent studies show that partial pulpotomy is a reliable preservative treatment approach for traumatized permanent anterior teeth with pulpal exposure. In our case, partial pulpotomy was indicated and successfully performed.

Factors influencing the successful VPT of traumatized permanent teeth

Pulp Exposure

When the pulp of a once-intact tooth is exposed as a result of trauma, it can often be assumed that the pulp is healthy and has the capacity to regenerate. This is definitely true in patients with no pre-existing pulp lesions due to caries or previous dental trauma, as long as there is no concomitant dislocation of the tooth. In a study by Cvek et al. in 1982, it was found that the size of pulpal exposure, ranging from 0.5 to 4.0 mm, after partial pulpotomy on traumatized teeth with complicated fractures, had no effect on healing frequency [8]. Clearly, it is not critical to treat complicated crown fractures immediately after trauma, but no research findings have yet evaluated the success rate of partial pulpotomy on teeth with pulpal exposure greater than 4 mm, and the prognosis has not yet been clearly determined. Primate models examining the tissue response after experimental exposure of pulps to the oral environment have shown the presence of inflammatory cells in the pulp at the exposure site. However, during the first hours of exposure, the tissue changes are more a reflection of damage resulting from mechanical injury, with negligible superficial inflammatory changes [9]. In our case, the size of the pulp exposure on tooth 11 was approximately between 2-3mm.

Bleeding

Partial pulpotomy requires the removal of approximately 2 mm of coronal pulp. Washing the pulp wound with sodium hypochlorite (0.5-5%) or chlorhexidine (0.2-2%) is recommended to enhance hemostasis and disinfection [10]. Cotton balls soaked in sodium hypochlorite can be gently applied. If the residual pulp is restored to a healthy level, bleeding will stop within 5 min. If hemostasis does not occur within this time, removal of the entire coronal pulp (complete pulpotomy) may be a consideration as a last option to preserve the vitality of the root pulp [9]. In our case the bleeding was controlled with cotton pellets moistened with 2.5% sodium hypochlorite and hemostasis is ensured during the 4-5 min.

Stage of Root Development

According to studies reported by Rao et al. in 2020, success rates varied from 93.75% to 97.4% for mature teeth and from 95.3% to 96.7% for immature teeth [11]. There was no significant difference between the success rates of partial pulpotomies for mature and immature teeth. Similarly, in a systematic review by Donnelly et al. in 2022, this study found that partial pulpotomy gave encouraging outcomes for mature and immature teeth with complicated crown fractures, with an overall success rate between 75% and 96.7% over a follow-up period of at least 12 months [12]. Likewise, other studies have demonstrated that partial pulpotomy after trauma is linked to very high success rates, particularly for immature teeth (90-100%), but also to high success rates between 70% and 100% for mature teeth [8]. In our case, the teeth are mature and they were successfully treated by partial pulpotomy.

Time Interval Between Trauma and Partial Pulpotomy Treatment

The time interval between trauma and partial pulpotomy treatment has been recorded with conflicting findings. In some case series, the time interval between trauma and treatment had no effect on treatment outcome, as long as the surviving pulp tissue was vital [8]. In contrast, a study by Cvek in 1993 reported that there was a difference in numeric success rates when the time interval before treatment was considered, with the success rate decreasing from 95.83% before 72 h to 87.5% after 72 h [13]. In contrast, in a clinical case of partial pulpotomy on 60 teeth with treatment intervals between 1 h and 90 days, that time was not a key factor in the regeneration of healthy pulp, based on a treatment success rate of 96.7%. However, most of the teeth evaluated were treated ≤100 h [8]. Other studies suggest that an interval of up to nine days between the damage and treatment may have minimal impact on the success of partial pulpotomies [5]. Another study by Rao et al. in 2020 described time intervals to treatment, which varied from less than 24 h to more than 72 h, and indicated that there was no significant difference between groups; nevertheless, they did not relate time intervals to overall success rate [11]. In our case, the time interval was 20 h between trauma on teeth 11 and 21 and the treatment by partial pulpotomy.

Concomitant Trauma

The presence of a concomitant trauma, defined as the presence of more than one lesion, would create a synergistic impact and therefore have a more adverse effect than a single lesion. In a study by Haikal et al. in 2020, the presence of a concomitant dislocation lesion was the only independent factor that significantly affected treatment outcome [14]. In accordance with the above, there was a significant rise in pulpal necrosis in mature teeth with crown fractures in combination with concussion or subluxation lesions. However, we must admit that the healing scenario is complicated and multifactorial, connected to the patient, trauma, or treatment factors [15]. In our case, no mobility, no luxation, or any concomitant dental injury was associated with both traumatized incisors.

Pulp Capping Materials

Several pulp capping materials have been used with varied physiomechanical characteristics such as seal ability, healing, antimicrobial effectiveness, biocompatibility, and bioactivity properties when brought into direct contact with inflamed pulp tissue. To evaluate the success rate of partial pulpotomy using different pulpotomy medicaments, the majority of evidence on comparisons between different partial pulpotomy medicaments was found in the literature comparing CH, MTA, Biodentine, and iRoot BP plus. Calcium hydroxide is well recognized as the standard pulp capping material, with a long history of clinical success principally linked to its excellent antimicrobial activity, biocompatibility, and ability to enhance calcified bridge formation [16]. Recent research has shown that this may be because CH provokes the release of bioactive molecules from the dentin matrix, including bone morphogenetic protein (BMP) and transforming growth factor beta one (TBF-β1), which stimulate pulp repair and dentin remineralization [1]. However, several disadvantages have been identified with the use of calcium hydroxide material, such as the lack of inherent adhesive properties, degradation, poor sealing ability, and a "tunnel effect" in the induced calcified bridge, which can cause inflammation or even cause necrosis due to intense leakage through these tunnels, allowing direct access of microorganisms to the pulp [1]. All these drawbacks have led to the innovation of new materials. Caprioglio et al. in 2014 recorded an 81.5% success rate using MTA for partial pulpotomy in 27 traumatized anterior teeth, MTA presents calcium oxide in the form of tricalcium silicate, dicalcium silicate, tricalcium aluminate, and bismuth oxide to make the material radiopaque [17]. Favorable properties of MTA comprise a significant reduction in pulp inflammation, thicker dentin, and less porosity. The advantages of MTA, such as low or non-existent solubility, high pH, excellent sealing, and biocompatibility properties, are well documented [2]. However, it has disadvantages such as tooth discoloration, long setting time, poor handling properties, and high cost. Studies have found that there is no statistical difference between the success rates of teeth treated with CH and those treated with MTA when performing partial pulpotomy on traumatized permanent teeth [2]. Likewise, another study by Haikal et al. in 2020 also found significantly greater crown discoloration with the use of MTA than with Biodentine, indicating that Biodentine can be considered a suitable alternative material providing better esthetic results, which is especially important when achieving partial pulpotomy on anterior permanent teeth [14]. The use of Biodentine for the partial pulpotomy of 48 traumatized anterior teeth, with a 91% success rate, was documented by Haikal et al. in 2020 [14]. Biodentine is considered a calcium silicate cement that was initiated as a "dentin replacement" material. It has the same clinical applications as MTA but with superior physicochemical properties, micro-mechanical anchorage, induction of effective calcium barrier formation, no tooth discoloration, faster setting time, and ease of handling [3]. In our case, the pulp capping material used on tooth 11 after performing partial pulpotomy was Biodentine.

Definitive Restoration

The time interval between VPT treatment and placing a definitive restoration varies, regardless of whether it's the first or subsequent visit. A retrospective study by Maguire et al. in 2000 demonstrated that immediate placement of a bonded definitive coronal filling augmented the mean vitality survival time of mature teeth [18]. Indeed, after a partial pulpotomy on a traumatized tooth, it is recommendable to fit a bonded definitive restoration as soon as possible, optimally during the same visit, in order to prevent unwanted coronal leaking and further pulpal infection [16]. In our case, the Biodentine was covered with a layer of resin-modified ionomer in the first visit and a directly bonded composite restoration was performed one week after.

Follow-up

Zanini et al. in 2016 stated that in order to evaluate the success of the treatment, a long follow-up period is essential, but when the follow-up period is very long, there may be failures due to bacterial leakage when the newly formed dentin bridge can no longer be able to protect the underlying pulpal tissue, resulting in irreversible pulpitis [19]. According to Fuks et al. in 1987, the risk of pulp necrosis or development of calcified metamorphosis can also be reported, hence the need for periodic follow-up and regular annual visits for patients. Yet the high incidence of long-term success supports the case for partial pulpotomy as the treatment of choice for fractured permanent incisors with pulp exposure [20]. Clinical and radiographic outcome criteria defining success are listed, such as absence of pain, positive pulpal sensitivity testing, soft tissue assessment, absence of mobility, absence of discoloration, radiographically visible dentin bridge formation, and absence of radiographic pathology.

As for our case, the two traumatized teeth have not shown any sign of pathology for a one-year follow-up. The successful management of the injured teeth and the performance of partial pulpotomy on tooth 11 were able to preserve the vitality of the pulp with positive pulp sensitivity testing and the absence of discoloration or pain, all these clinical and radiographical signs led to a good prognosis.

## Conclusions

Maintenance of pulp vitality in injured mature teeth should be a treatment goal. The high rates of success that have been recorded for partial pulpotomy indicate that this procedure should be regarded as the treatment of choice for mature teeth that have suffered complicated crown fractures. The most recently available evidence suggests that the bioactive alternative biomaterials, Biodentine and iRoot BP, are both associated with high clinical and radiographic success and are deemed an appropriate VPT biomaterial when a partial pulpotomy is performed.

The results of our case fit within the existing literature, complete success both clinically (vitality and esthetics) and radiographically (absence of pathological findings) was observed for the mature traumatized tooth "11" after a follow-up of 12 months. On this basis, pulp treatment is strongly recommended using Biodentine as an excellent pulp dressing for the treatment of complicated crown fractures.
